# Predicting Malignant Transformation in Oral Epithelial Dysplasia: A Systematic Comparison of Artificial Intelligence‐Based Risk Models and Pathologist‐Based Microscopy

**DOI:** 10.1111/jop.70168

**Published:** 2026-07-23

**Authors:** Diele Carine Barreto Arantes, Lucas Monteiro Delgado, Marília de Marco Pinto Paiva dos Santos, Saman Warnakulasuriya, Marcelo Sperandio

**Affiliations:** ^1^ Oral Medicine and Pathology, Faculdade São Leopoldo Mandic Research Institute, Rua Dr. José Rocha Junqueira Campinas São Paulo Brazil; ^2^ Modelo Clinic, Oral and Digestive Medicine Belo Horizonte Minas Gerais Brazil; ^3^ Faculty of Medicine Federal University of Minas Gerais Belo Horizonte Minas Gerais Brazil; ^4^ WHO Collaborating Centre for Oral Cancer and Faculty of Dentistry, Oral and Craniofacial Sciences King's College London London UK

**Keywords:** artificial intelligence, malignant transformation, oral epithelial dysplasia, oral leukoplakia, risk prediction models

## Abstract

**Background/Aims:**

Risk prediction models (RPMs) based on histopathological analysis of oral epithelial dysplasia (OED) are increasingly used to stratify patients with oral potentially malignant disorders (OPMDs) and support personalized management. This systematic review and meta‐analysis evaluated artificial intelligence (AI)‐based models compared with conventional human microscopy for predicting malignant transformation (MT).

**Methods:**

A total of 32 studies published between 1998 and 2026 were included (9 AI‐based and 23 human‐based), focusing on OED in oral leukoplakia (OL), erythroleukoplakia, and erythroplakia. AI approaches addressed prediction or prognostic tasks using machine learning (ML), deep learning (DL), or hybrid methods, with multilayer perceptron and random forest among the most frequently used algorithms.

**Results:**

Meta‐analysis demonstrated strong discriminative performance for AI models (pooled odds ratio [OR] = 12.64; 95% CI: 5.65–28.26; *I* ^2^ = 57%). Human evaluation also confirmed a significant association between OED and MT (OR = 3.42; 95% CI: 2.07–5.68), with substantial heterogeneity (*I* ^2^ = 85%). AI‐based models showed higher specificity and more consistent performance, while sensitivity remained comparable to human assessment.

**Conclusions:**

Overall, AI‐based RPMs demonstrate promising and more reliable discriminative ability, primarily by improving decision precision rather than detection rates. However, further standardization, external validation, and larger multicentric datasets are required for clinical implementation.

## 1 Introduction

Despite substantial advances in oncological management, the 5‐year survival rate for patients with oral cancer remains approximately 50%–60%, and mortality continues to increase annually by 1%–2% across both sexes [1, 2]. Treatment‐related toxicities further compromise quality of life, and head and neck malignancies are associated with one of the highest suicide risks among all cancer types, accounting for up to 20% of suicides in oncological populations [3].

Approximately 90% of oral cancers are squamous cell carcinomas of epithelial origin [1]. Oral squamous cell carcinoma (OSCC) is often preceded by a precancerous phase in which genetically altered keratinocytes undergo clonal expansion, resulting in histopathological and clinical changes termed oral potentially malignant disorders (OPMDs) [4, 5, 6]. OPMDs include leukoplakia, erythroplakia, erythroleukoplakia, actinic cheilitis, oral submucosal fibrosis, reverse smoker's palate, oral lichen planus, lichenoid reactions, graft‐versus‐host disease, oral lupus erythematosus, and rare inherited conditions such as dyskeratosis congenital [7].

Leukoplakia is the most frequent OPMD, predominantly affecting men with chronic tobacco and/or alcohol use, and it is defined as a “white plaque of questionable risk having excluded (other) known diseases or disorders that carry no increased risk for cancer” [8, 9]. Leukoplakia precedes OSCC in nearly 67% of cases, though malignant transformation (MT) rates vary widely, from 0.13% to 40.8% [10, 11]. Risk factors for MT include female sex, age > 50 years, nonhomogeneous clinical appearance, severe oral epithelial dysplasia (OED), and high‐risk anatomical sites such as the tongue, floor of the mouth, ventral tongue, buccal mucosa, and labial mucosa [8, 12, 13, 14]. Histologically, leukoplakia may present as hyperkeratosis, dysplasia, or carcinoma, with dysplasia reflecting chromosomal instability and a transitional state toward MT [6].

OED is the principal prognostic marker for MT in OPMDs, whereas histopathological evaluation of biopsy specimens remains the diagnostic gold standard. However, inter‐ and intra‐observer variability continues to limit diagnostic and prognostic reliability [8, 15]. The World Health Organization (WHO) currently endorses two grading systems: the conventional three‐tier scheme (mild, moderate, and severe), which is based on the extent of architectural and cytological atypia across epithelial layers [16]; and the binary system, proposed by Kujan et al. [17], which stratifies lesions into low‐risk and high‐risk categories, the latter defined by at least four architectural and four cytological abnormalities [9, 17, 18, 19].

Risk prediction models (RPMs), based on statistical methods and machine learning (ML), are increasingly applied in oncology to stratify patients and guide individualized treatment [20, 21]. High‐grade OED has been consistently associated with increased MT risk and poorer disease‐free survival [6], yet grading still depends on manual histopathological assessment, which is subjective and prone to variability [8, 15]. Deep learning (DL), particularly artificial neural networks, has demonstrated strong potential in automating histopathological image analysis of whole slide images (WSIs) and patches [13, 20, 21, 22, 23]. Recent studies have applied automated approaches to classify OPMDs and OED from clinical and histological images [13, 23, 24, 25], complementing earlier reviews [26, 27] but underscoring the need for a more comprehensive synthesis.

Thus, this systematic review and meta‐analysis aimed to evaluate the performance of artificial intelligence (AI)‐based RPMs for histopathological assessment of OED and leukoplakia, erythroleukoplakia, and erythroplakia, compared with conventional microscopy. Specifically, we addressed four key research questions: (1) Which AI‐based RPMs achieve the highest accuracy in predicting MT? (2) How do AI‐based RPMs compare with conventional hematoxylin and eosin (H&E)‐based evaluation? (3) Which ML and DL techniques have been applied in automated OED assessment? and (4) What classification and segmentation tasks are used in AI‐based reporting?

## 2 Materials and Methods

### 2.1 Protocol and Registration

This systematic review and meta‐analysis were conducted in accordance with the Cochrane Handbook for Systematic Reviews of Diagnostic Test Accuracy and reported following the Preferred Reporting Items for Systematic Reviews and Meta‐Analyses of Diagnostic Test Accuracy Studies (PRISMA‐DTA) guidelines. The study protocol was registered in the International Prospective Register of Systematic Reviews (PROSPERO) under the registration number CRD42024608333.

### 2.2 PICOS Framework

The study was structured according to the PICOS framework:

- *P (Population)*: OED in OPMD
- *I (Intervention)*: AI‐based approaches
- *C (Comparator)*: Conventional H&E microscopy
- *(Outcomes)*: Performance of RPMs
- *S (Study design)*: Observational studies and comparative studies

### 2.3 Eligibility Criteria

Studies were eligible for inclusion if they met all of the following criteria: (1) primary morphological analysis of H&E‐stained histopathological images of OED, with or without clinical data; (2) AI studies with application of ML or DL methods for automated histopathological assessment; and (3) reporting performance metrics of RPMs.

Studies were excluded if they had any of the following conditions: (1) focused solely on clinical images; (2) addressed histopathology of OPMDs other than leukoplakia, erythroleukoplakia, or erythroplakia (e.g., OSCC, lichen planus, or proliferative verrucous leukoplakia); (3) use of non‐H&E modalities as the primary data source (e.g., spectroscopy, confocal microscopy, or narrow‐band imaging); (4) animal studies; (5) case reports, books, social media content, meta‐analyses, or narrative reviews.

To harmonize variability in terminology across the included studies, reported categories were mapped into comparable analytical strata. Lesions described as epithelial hyperplasia, hyperkeratosis/hyperparakeratosis, and acanthosis were classified as non‐dysplastic. MT was defined as the development of OSCC at the same or an adjacent site during the follow‐up period specified in each study. Human studies were grouped according to OED classification as follows: stage‐based classification I–IV [28]; three‐tier grading system—mild, moderate, and severe dysplasia [16]; two‐tier grading system [17]; and differentiated dysplasia [29].

### 2.4 Search Strategy and Study Selection

A systematic search was performed across PubMed, Embase, and the Cochrane Library, covering all literature up to August 22, 2024, and updated on March 17, 2026. Given the AI focus of this review, additional searches were conducted in computer science databases, including IEEE Xplore and the ACM Digital Library. The search strategy for each database is detailed in Table S1. We also searched the reference lists of included studies and previous systematic reviews and meta‐analyses to identify additional relevant studies.

Two reviewers (D.C.B.A. and M.S.) independently performed the searches and screened results using Rayyan Software (Qatar Computing Research Institute, Qatar Foundation). After removing duplicates and studies not aligned with the review focus, full‐text screening was conducted to determine final inclusion.

### 2.5 Data Extraction

Two reviewers (D.C.B.A. and M.S.) independently extracted data into a standardized form, including:

1. *General study information*: Authors, year of publication, country, study type, data source(s), and main findings
2. *AI‐related information*: ML/DL architectures used for OED classification or segmentation, model characteristics, input image types (WSI or patches), training/validation/testing approaches, and RPM performance metrics
3. *Comparative analysis*: RPM performance of conventional H&E microscopy for benchmarking against AI models

### 2.6 Risk of Bias Assessment

The risk of bias of the included studies was independently assessed by two reviewers (D.C.B.A. and M.S.) using the *Joanna Briggs Institute (JBI) Critical Appraisal Checklist for Diagnostic Test Accuracy Studies*. This tool evaluates methodological quality across nine domains (Q1–Q9), including patient selection, index test, reference standard, and flow and timing.

Each item was scored as “Yes” (low risk of bias), “No” (high risk of bias), or “Unclear.” The results were synthesized in tabular format, and disagreements were resolved through discussion. The overall assessment of each study was used to generate a visual summary of quality using bar plots and a quality plot diagram.

### 2.7 Statistical Analysis

The sensitivity and specificity of each RPM were calculated from the extracted counts of true positives (TPs), false positives (FPs), true negatives (TNs), and false negatives (FNs), with 95% confidence intervals (CIs) subsequently computed. In studies using a three‐tier classification without a no‐dysplasia control group, mild OED was considered the reference (negative) category, while moderate and severe dysplasia were grouped together. In studies including a no‐dysplasia group, all grades of dysplasia (mild, moderate, and severe) were combined and compared against the no‐dysplasia category. A *p* < 0.05 was considered statistically significant for overall effect estimates. All meta‐analyses were performed using DerSimonian and Laird random‐effects models to account for between‐study heterogeneity.

Heterogeneity was assessed using Cochran's *Q* test and the *I* ^2^ statistic, with *p* < 0.10 and *I* ^2^ > 25% indicating significant heterogeneity, in accordance with Cochrane recommendations. Additionally, between‐study variability was visually explored using summary receiver operating characteristic (SROC) curves, where the dispersion of individual study estimates and the size of the confidence and prediction regions reflect the degree of heterogeneity.

## 3 Results

### 3.1 Study Selection

A total of 7047 records were identified through electronic databases, including PubMed (*n* = 2103), Embase (*n* = 3968), Cochrane (*n* = 961), IEEE Xplore (*n* = 3), and ACM Digital Library (*n* = 12). After removing 1760 duplicates, 3527 records were screened, of which 3471 were excluded based on title and abstract.

A total of 56 full‐text reports were assessed for eligibility, with 34 subsequently excluded for reasons such as incomplete data (*n* = 10), clinical image analysis only (*n* = 4), diagnosis only (*n* = 3), retracted studies (*n* = 2), database‐only reports (*n* = 1), studies using unrelated techniques (*n* = 8), focus on other OPMDs (*n* = 4), and limited OED stratification (*n* = 2).

Two additional records were identified through citation searching, and eight were included following the updated search. Ultimately, 32 studies were included in the systematic review (Figure 1).

**FIGURE 1:**
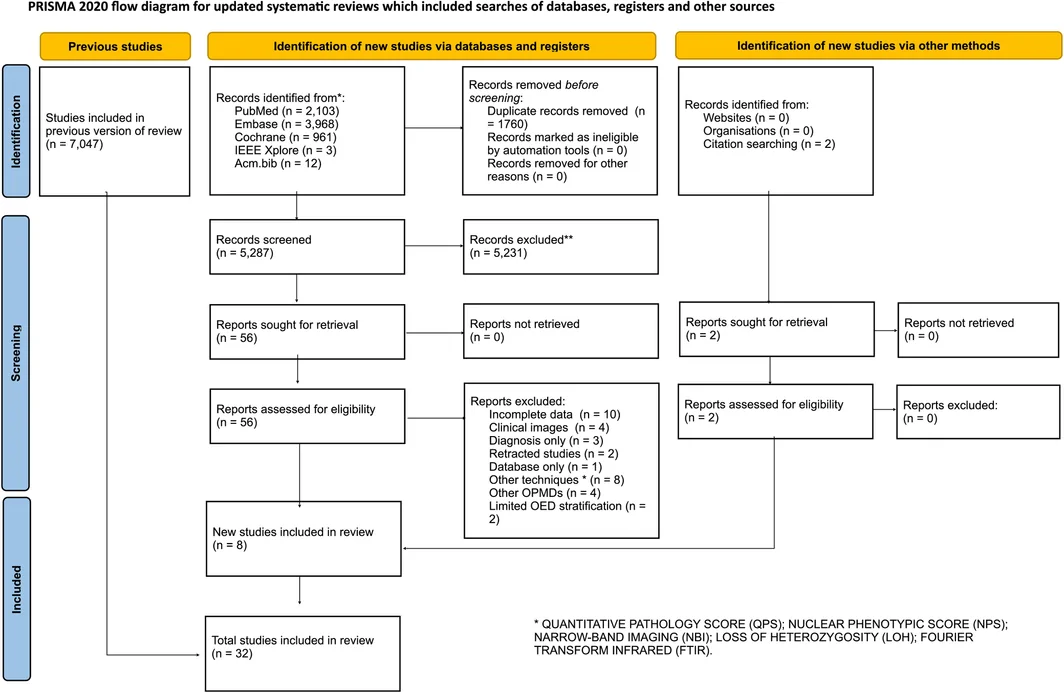
PRISMA 2020 flow diagram illustrating the selection process for the 32 studies included in the systematic review.

### 3.2 Study Characteristics

The 32 studies included in this review were published between 1998 and 2026, with 27 (84.3%) published from 2020 onwards. Most human‐based studies were conducted in Europe (*n* = 13; 56.5%), followed by South America (*n* = 3; 13.0%), Asia (*n* = 3; 13.0%), North America (*n* = 2; 8.6%), and Oceania (*n* = 2; 8.6%). A similar distribution was observed for AI‐based studies, with most conducted in Europe (*n* = 4; 44.4%) and South America (*n* = 3; 33.3%), followed by North America (*n* = 2; 22.2%) and Oceania (*n* = 2; 22.2%).

Conventional human‐based OED assessment was performed in 23 studies [12, 28, 29, 30, 31, 32, 33, 34, 35, 36, 37, 38, 39, 40, 41, 42, 43, 44, 45, 46, 47, 48, 49], of which only one was prospective [28] (Table 1). This group included 5733 cases, comprising 3360 (58.6%) OL, 29 (0.5%) erythroleukoplakia, 15 (0.2%) erythroplakia, and 2329 (40.6%) OED not otherwise specified. The mean rate of MT was 29.25% (range: 1.49%–62%), with variable minimum follow‐up periods (5–72 months).

**TABLE 1:** Summarized characteristics of human‐based studies.

OED classification	Study	Year	Country	Study type	Lesion type (*n*)	Sample size (*n*)	Follow‐up minimum (months)	Malignant transformation (%)	TN/FN/TP/FP
Stage	Schepman et al. [28]	1998	The Netherlands	Observational follow‐up study	OL (166)	Stages I–III low risk (62), Stage IV high risk (47)	6	12.94	59/3/11/36
	Brouns et al. [30]	2014	The Netherlands	Retrospective cohort study	OL (144)	No dysplasia and mild/Stages I and II (68), Moderate/Stage III (51) Severe/Stage IV (25)	12	11	64/4/12/64
WHO/traditional/3 tiers (with no dysplasia group)	Zaini et al. [31]	2018	UK/Malaysia	Retrospective cohort study (OED grade + DNA ploidy analysis)	White lesion (208), Speckled (15), Red (5)	No dysplasia (121), Mild (47), Moderate (30), Severe (21)	6	10.5	120/1/23/98
	Shearston et al. [32]	2019	Australia	Retrospective cohort study	OL (202)	No dysplasia (152), Mild (30), Moderate (8), Severe (5), Dysplasia NOS (6)	18	1.49	150/2/1/48
	Chaturvedi et al. [33]	2020	USA	Retrospective cohort and case‐cohort study	OL (489)	No dysplasia (422), Mild (42), Moderate (16), Severe (9)	12	2.2	384/38/33/34
	Bagan et al. [34]	2022	Spain	Retrospective cohort study of surgical cases	OL (224)	No dysplasia (143), Mild (57), Moderate (22), Severe (2)	9	11.6	132/11/15/66
	Tovaru et al. [35]	2023	Romania	Retrospective study	OL (120)	No dysplasia (75), Mild (34), Moderate (9), Severe (2)	12	7.5	73/2/7/38
WHO/traditional/3 tiers (without no dysplasia group)	Hankinson et al. [36]	2021	UK	Cohort study on OED transformation risk	OED (150)	Mild (77), Moderate (49), Severe (24)	NR	9.33	75/2/9/64
	Ellonen et al. [37]	2023	Finland	Retrospective cohort	OED (681)	Mild (425), Moderate (198), Severe (22)	72	62	401/24/25/195
	Yasothkumar et al. [38]	2025	India	Retrospective	OED (46)	Mild (13), Moderate (16), Severe (17)	36	8	13/0/3/30
WHO/traditional/3 tiers (without no dysplasia group) and BINARY, 2 tiers	Dost et al. [39]	2014	Australia	Retrospective cohort study	OED (383)	Mild (221), Moderate (85), Severe/CIS (55); Low risk (221), High risk (140), Dysplasia NOS (22)	6	4.7	212/9/9/153 (3 tiers); 212/9/7/133 (2 tiers)
	Mahmood et al. [40]	2022	UK/USA/Australia	Retrospective pathology study	OED (109)	Mild (34), Moderate (48), Severe (27); Low risk (73), High risk (36)	60	18	33/1/19/56 (3 tiers); 65/8/12/24 (2 tiers)
	Mahmood et al. [41]	2023	UK and Brazil	Retrospective multicenter cohort	OED (196)	No dysplasia (25), Mild (58), Moderate (68), Severe (70); Low grade (76) High grade (120)	60	25	55/3/36/102 (3 tiers)
	Cai et al. [42]	2024	China	Retrospective cohort	OL (201)	Mild (121), Moderate (51), Severe (29); Low risk (122), High risk (79)	NR	29.35	30/91/29/51 (3 tiers); 30/92/29/50 (2 tiers)
	Mahmood et al. [43]	2025	UK and Brazil	Multicentric (four different centers) retrospective prognosis prediction	OED (104)	Mild (28), Moderate (41), Severe (33); 6 point‐Low grade (37), High‐grade (65)	36	28	—
	Ellonem et al. [44]	2025	Finland	Retrospective histopathological reevaluation analysis + clinical data	OED (612)	Mild (287), Moderate (144), Severe (17); Low‐grade (397), High‐grade (75); Dysplasia NOS (24)	72	7.8	367/30/7/68 (2 tiers)
WHO/traditional/3 tiers (with no dysplasia group) and BINARY, 2 tiers	Jayasooriya et al. [45]	2020	Sri Lanka	Retrospective cohort study	OL (93)	No dysplasia (20), Mild (34), Moderate (20), Severe (19); Low risk (34), High risk (39)	10	7.52	20/0/7/66 (3 tiers); 33/1/6/33 (2 tiers)
	Sperandio et al. [12]	2023	Brazil	Retrospective histopathological and clinical study	OL (878)	No dysplasia (262); Mild (259), Moderate (265), Severe (92); Low risk (625), High risk (253)	36	4.14	257/5/30/586 (3 tiers); 610/15/20/233 (2 tiers)
	Darling et al. [46]	2023	Canada/UK	Retrospective cohort study using biomarker	OED (48)	No dysplasia (2); Mild (5), Moderate (21), Severe (18); Low grade (9), High grade (35)	5	63	1/1/29/15 (3 tiers); 4/5/24/11 (2 tiers)
	Rodrigues et al. [47]	2025	Brazil	Retrospective cohort	OED (195 of leukoplakia 171, erythroplakia 10 and erythroleukoplakia 14)	No dysplasia (84); Mild (70), Moderate (29), Severe (12); Low grade (88), High grade (23); Low grade (70), High grade (41); Low grade cutoff (74) High grade cutoff (37)	5	16.41	82/2/32/79 (3 tiers); 68/20/12/11 (2 tiers 2017); 60/10/22/19 (2 tiers 2022); 62/12/20/17 (2 tiers 2022 cutoff)
Binary, 2 tiers	Cerqueira et al. [48]	2021	Brazil	Multicenter retrospective cohort study	OL (148)	No dysplasia (36), Dysplasia (112); Low risk (90); High risk (58)	NR	8.8	35/1/12/100 (binary); 86/4/9/49 (2 tiers)
Differentiated dysplasia	Brouns et al. [49]	2023	The Netherlands	Retrospective multicenter study	OL (140)	Stages I and II (no dysplasia 46), Stage III (mild or moderate 45), Stage IV (severe or DD 49)	12	22.14	46/0/44/50
	Wils et al. [29]	2023	The Netherlands	Retrospective study on DD in OL	OL (176)	No dysplasia (76), Any dysplasia (100); Mild (35), Moderate (11), Severe (13); DD (117), classic OED (59), No DD (126), DD (50)	12	19	73/3/30/70

Nine retrospective studies adopted AI‐based approaches [13, 50, 51, 52, 53, 54, 55, 56, 57], including a total of 1813 cases, of which 381 (21.0%) were OL, and 1432 (79.0%) were OED not otherwise specified (Table 2). AI models were applied for predictive (*n* = 6; 66.7%) or prognostic (*n* = 3; 33.3%) tasks. Among these, three studies used ML approaches [54, 56, 57], three used DL methods [13, 52, 55], and three combined both approaches [50, 51, 53]. The algorithms were heterogeneous, although the multilayer perceptron (MLP) and random forest were the most frequently used. All AI models were based on H&E‐stained WSI or image patches, with or without clinical data. However, several studies incorporated additional data sources, including immunohistochemistry [52], lymphocytic infiltration analysis [51, 53, 55], architectural and cytological features [54], and DNA content [56]. In contrast, most human‐based studies relied exclusively on H&E‐stained histopathological slides, with only two studies incorporating complementary data such as immunohistochemistry [46] and DNA content analysis [31].

**TABLE 2:** Summarized characteristics of AI‐based studies.

Study	Year	Country	Model family	Algorithm/architecture	Task	Input data (*n*)	Best‐performing model	Training/validation/test	TN/FN/TP/FP
Bashir et al. [50]	2020	UK and Pakistan	DL + ML	DeepLab v3 + Random Forest	Prediction	WSI H&E OED (35)	2 Tiers (DeepLab v3): F1—54%, SEN—57%, PREC—23%, Accuracy—57.5%	85% training/15% test	—
Zhang et al. [13]	2023	USA and Brazil	DL	CNN	Prognostic	WSI H&E OL‐OED (62)	OMRS model (modified Inception V3 architecture): HR—4.52, Accuracy—95.4%	Training, validation and test with images of non‐OED and OSCC	23/8/18/13
Bashir et al. [51]	2023	UK	ML + DL	IDaRS (ResNet‐34), MLP, Attention‐MIL, CLAM, ResNet‐50, DenseNet, ViT	Prognostic	WSI H&E OED + digital lymphocytic score (137)	Combined data (IDaRS): F1—69%, AUROC ~78% 3 Tiers: C‐index—68% 2 Tiers: C‐index—68%	Three different stratified fivefold cross‐validations	≈80/≈15/≈44/≈24
Cai et al. [52]	2023	China	DL	ResNet50, ResNet101, InceptionV3, DenseNet121	Prediction	WSI H&E OED + IHC (759)	Pathomics‐based model (Resnet50 + LightGBM): AUC—89.9%, SEN—83.8%, SPEC—80.2%, PREC—45.2%	Training (64.4%), validation (25.8%), test (9.7%).	≈43/0/11/20
Shephard et al. [53]	2024	UK	ML + DL	MLP + HoVer‐Net+	Prediction	WSI H&E OED + peri‐epithelial and intra‐epithelial lymphocytes analysis (282)	OMT score (MLP + HoVer‐Net+): F1—69%, AUROC—75%, SENS—92%, SPEC—40%, HR—8.48, C‐index—60%	Internal training/cross‐validation and independent two external validation	108/7/35/43
Soares et al. [54]	2025	Brazil	ML	Random Forest	Prediction	WSI H&E OL‐OED + clinical data + architectural and cytologic analysis (99)	Combined data (excluding all attributes with an MDG < 1): SEN—96%, SPEC—67%, PREC—96%, Accuracy—93%	Training (70%), testing (30%)	—
Shephard et al. [55]	2025	UK	DL	Trans‐UNet + CNN‐based HoVer‐Net+	Prognostic	WSI H&E OED + clinical data + intra‐epithelial lymphocytes analysis (219)	Combined data (Trans‐UNet + HoVer‐Net + and Cerberus): HR—12.14, C‐index—82% 3 Tiers (mild vs. moderate/severe): HR—8.06, C‐index—71.6% 2 Tiers: HR—8.20, C‐index—75.8%	Pretrained model	—
Pontes et al. [56]	2026	Brazil and UK	ML	MLP	Prediction	WSI H&E OL‐OED + clinicopathological data + DNA content (97)	Combined data (MLP): F1—74%, AUC—85%, SEN—73%, SPEC—96%, PREC—78%, Accuracy—91%	Cross‐validation, repeating training and testing five times with alternating datasets	71/5/13/3
Pedroso et al. [57]	2026	Brazil	ML	Logistic Regression, Random Forest, XGBoost, MLP, Decision Tree	Prediction	WSI H&E OL‐OED + clinical data (123)	Combined data (MLP): F1—55%, AUC—77%, SEN—70%, SPEC—94%, PREC—46%, Accuracy—82%	Nested cross‐validation	—

Among human‐based studies, eight applied the WHO three‐tier grading system, either with [31, 32, 33, 34, 35] or without a non‐dysplastic category [36, 37, 38]. One study used a binary grading system [48], while 10 studies applied both approaches, either with [12, 45, 46, 47] or without a non‐dysplastic category [39, 40, 41, 42, 43, 44]. Four studies adopted alternative classification systems, including stage‐based criteria [28, 30, 49] or differentiated dysplasia [29, 49].

The performance metrics of AI‐based RPMs varied substantially across studies. Four studies employed cross‐validation strategies, and only one study reported independent external validation [53]. Notably, one study included non‐OED and OSCC images in the training, validation, and testing datasets [13]. The F1‐score was explicitly reported in five of the nine studies. In several cases, the parameters required to derive the F1‐score were not available, limiting the possibility of standardizing performance comparisons across studies.

### 3.3 Risk of Bias Assessment

Across the 32 studies included, the risk of bias analysis using the JBI checklist (Q1–Q9) is presented in Figure 2. Human‐based studies demonstrated predominantly low risk of bias across most domains, with some concerns mainly related to outcome definition and follow‐up reporting (Q4–Q7). In contrast, AI‐based studies showed greater variability, particularly in domains related to model development and validation, with a higher frequency of high or unclear risk in Q5 and Q8, reflecting limitations in sample adequacy and lack of external validation.

**FIGURE 2:**
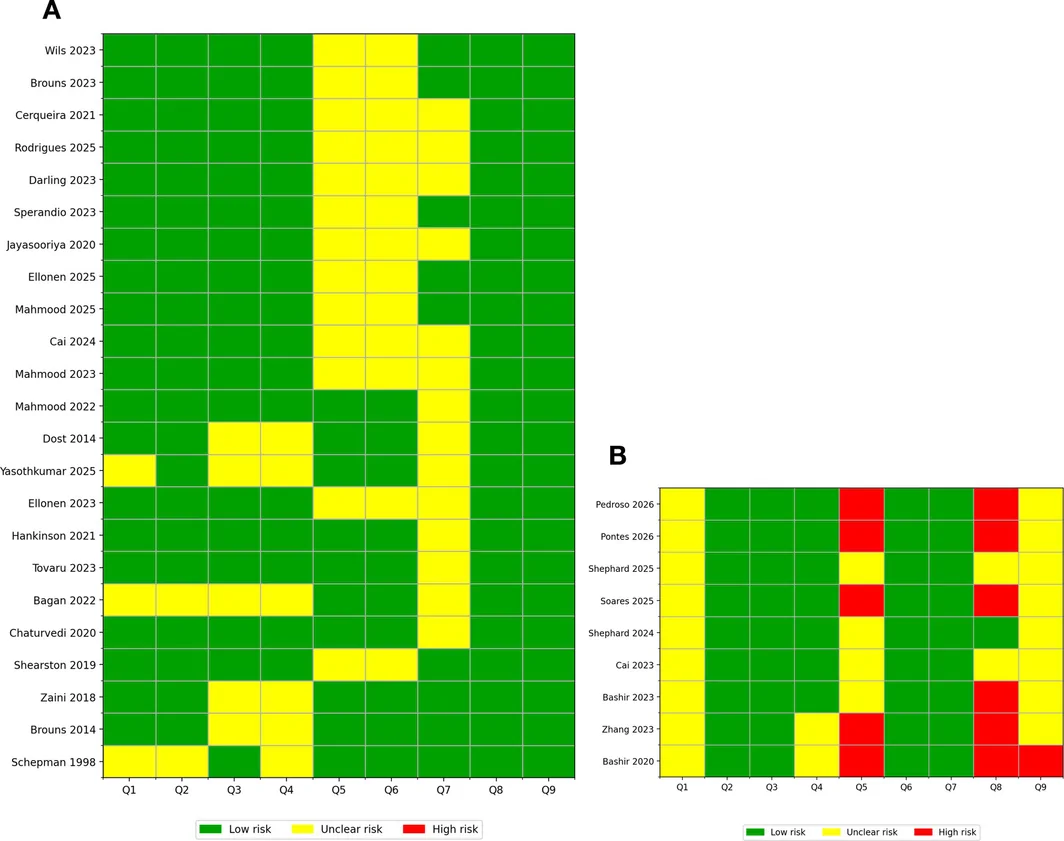
Risk of bias assessment across the 32 included studies using the JBI Checklist for Diagnostic Test Accuracy. (A) Human‐based studies (*n* = 23). (B) AI‐base studies (*n* = 9). Each row represents an individual study, and each column corresponds to one of the nine methodological domains (Q1–Q9). Green indicates low risk of bias, yellow indicates unclear or moderate risk, and red indicates high risk.

### 3.4 Quantitative Evaluation

Figure 3 presents the pooled meta‐analysis of 22 human‐based studies. In the subgroup analysis using a two‐tier classification, the combined OR for MT was 2.66 (95% CI: 1.27–5.61). Similarly, studies applying a three‐tier classification showed a pooled OR of 3.55 (95% CI: 1.70–7.41). For studies evaluating differentiated dysplasia, the pooled OR was 20.64 (95% CI: 2.66–160.41), although based on a limited number of studies. When all studies were combined, OED was associated with a significantly increased risk of MT (OR = 3.42; 95% CI: 2.07–5.68). Substantial heterogeneity was observed across studies (*I* ^2^ = 85%), and no significant differences were detected between subgroup estimates (*p* = 0.183). Consistently, stratified analyses by dysplasia grade revealed a stepwise increase in MT risk, with pooled ORs of 3.36 (95% CI: 1.85–6.10) for mild, 7.62 (95% CI: 3.91–14.85) for moderate, and 18.98 (95% CI: 10.25–35.14) for severe dysplasia, with decreasing heterogeneity as severity increased (Figure S1).

**FIGURE 3:**
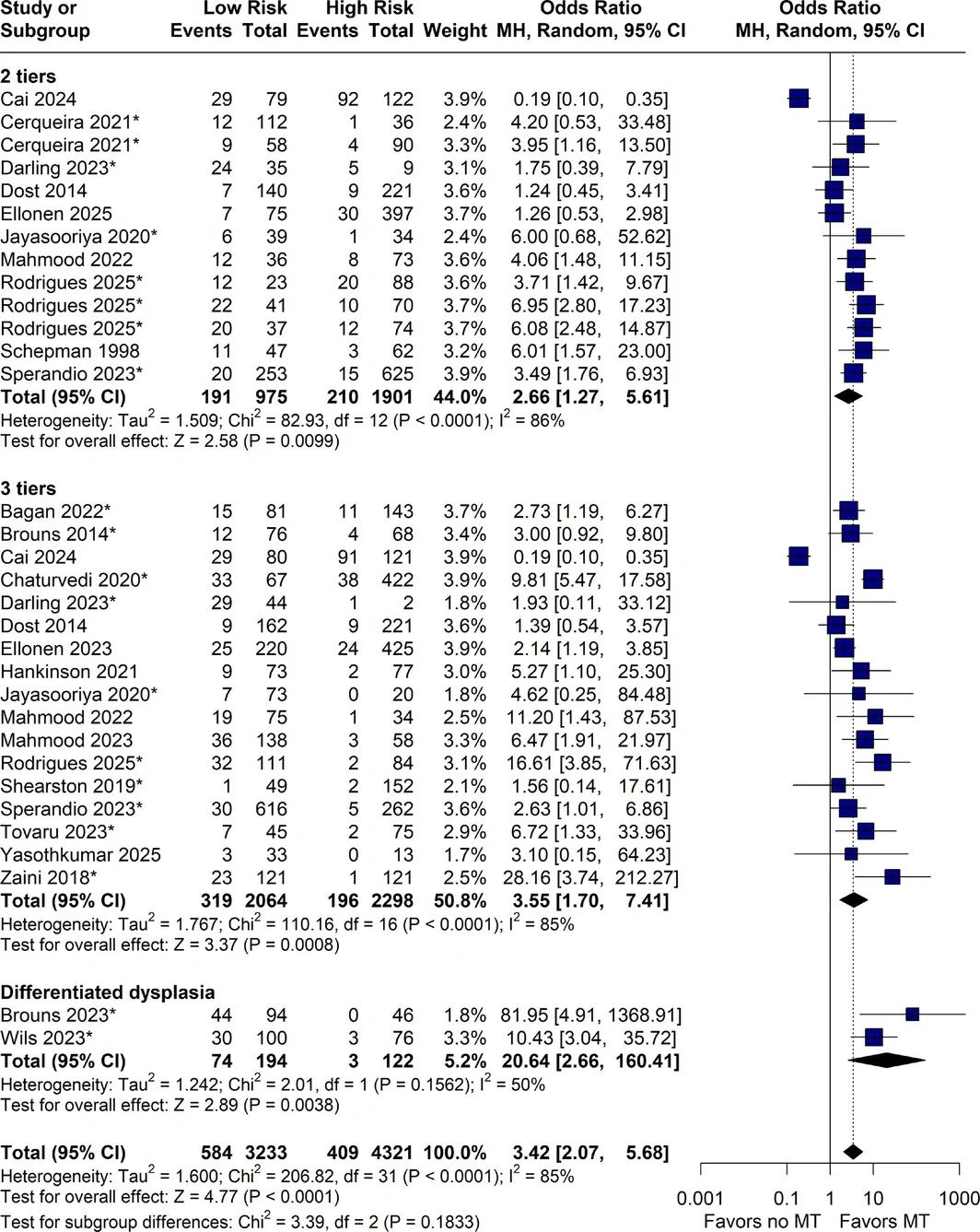
Forest plot of human‐based studies assessing the risk of MT in OED, with subgroup analyses (two‐tier, three‐tier, and differentiated dysplasia). Pooled OR = 3.42 (95% CI: 2.07–5.68; *I* ^2^ = 85%). Random‐effects model; squares represent individual studies and diamonds the pooled estimates. Asterisks indicate studies that included a no‐dysplasia control group.

The meta‐analysis of five AI‐based RPMs for OED (Figure 4) demonstrated a significantly higher risk of MT in the high‐risk group compared with the low‐risk group (pooled OR = 12.64; 95% CI: 5.65, 28.26; *p =* 0.0518; *I* ^2^ = 57%).

**FIGURE 4:**
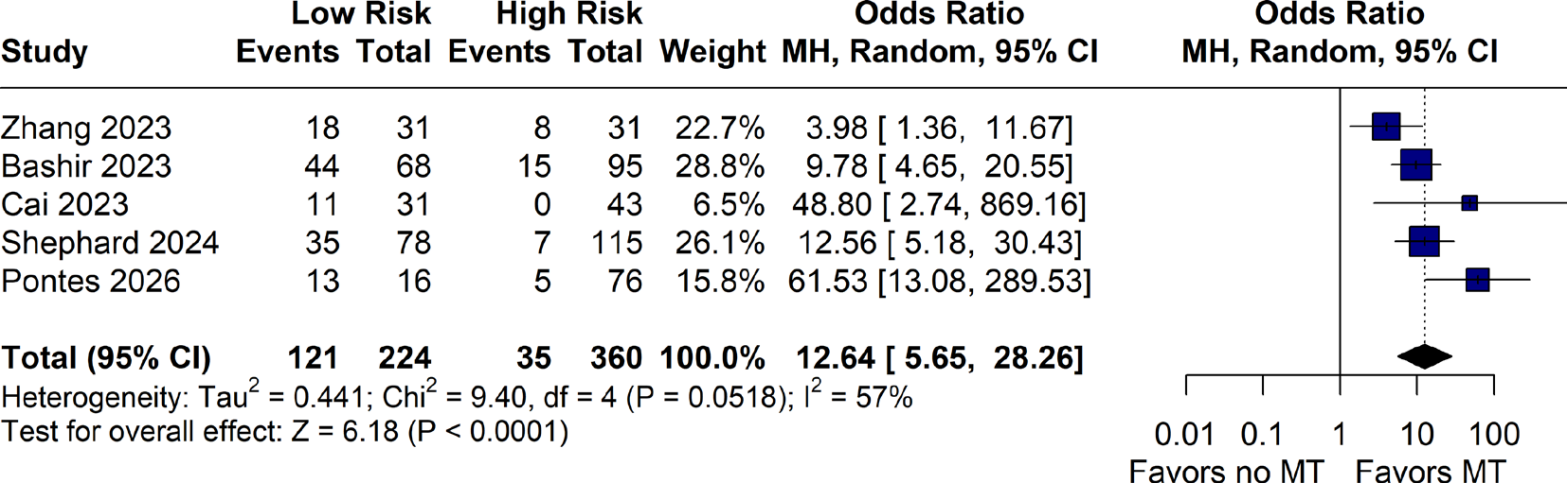
Forest plot of AI‐based studies assessing the risk of MT in OED. ORs with 95% confidence intervals (CIs) were calculated using a random‐effects model. Squares represent individual study weights, and the diamond represents the overall effect size with its 95% CI.

### 3.5 Sensitivity and Specificity

Figure 5 summarizes the pooled sensitivity and specificity of both human and AI‐based RPMs for MT in OED. AI‐based models demonstrated higher overall performance, with pooled sensitivity of approximately 0.78 (95% CI = 0.72–0.84) and a specificity of approximately 0.70 (95% CI = 0.63–0.77). In comparison, human‐based microscopy showed slightly lower pooled sensitivity of approximately 0.74 (95% CI = 0.68–0.80) and notably lower specificity of approximately 0.56 (95% CI = 0.48–0.64).

**FIGURE 5:**
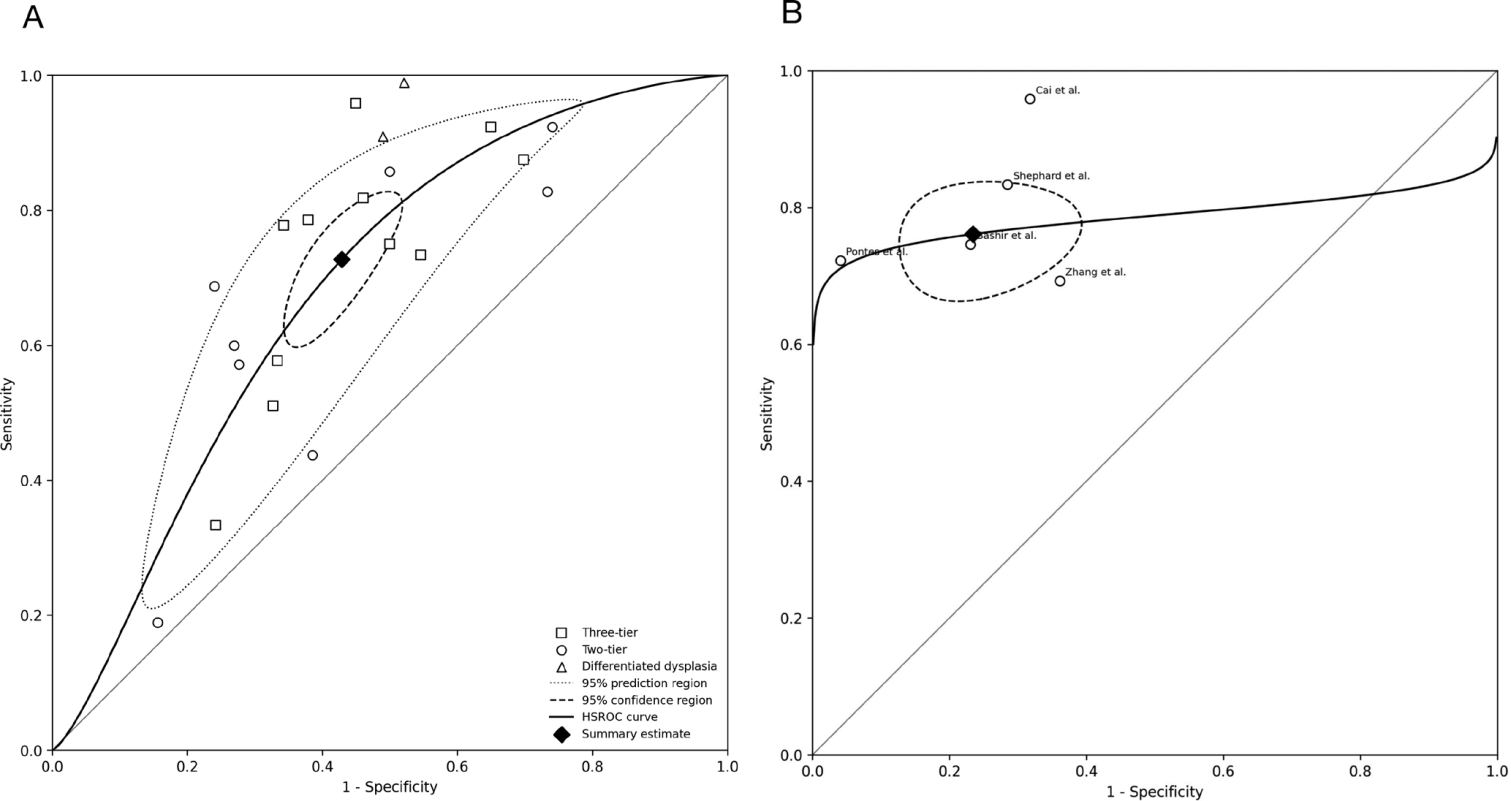
Summary receiver operating characteristic (SROC) curves for studies evaluating the prediction of MT in OED. (A) Human‐based studies, including two‐tier, three‐tier, and differentiated dysplasia classification systems, excluding the studies by Cai et al. [42] and Schepman et al. [28]. (B) AI‐based studies. Each point represents an individual study; the solid curve indicates the SROC, and the dashed ellipse represents the 95% confidence region around the summary estimate.

### 3.6 Heterogeneity and Influence Analysis

Substantial heterogeneity was observed in the human dataset, particularly for specificity, as reflected by the wide dispersion of individual study estimates and broad confidence intervals in the forest plots. Sensitivity estimates also showed moderate variability across studies. Two human‐based studies were excluded from this analysis [28, 42] due to marked discrepancies compared with the remaining studies. In contrast, AI‐based studies demonstrated lower between‐study variability, with more consistent effect estimates and narrower confidence intervals, especially for specificity. Overall, these findings indicated that heterogeneity in human‐based classification was more pronounced and widely distributed, whereas AI‐based models showed a more homogeneous performance.

### 3.7 Residual Heterogeneity

After accounting for differences between AI‐ and human‐based approaches, residual heterogeneity was reduced across outcomes, although moderate variability persisted, particularly for specificity. These findings suggest that part of the between‐study heterogeneity is explained by differences in classification methodology, while other factors, such as study design and population characteristics, may also contribute.

### 3.8 Bivariate Diagnostic Performance

The bivariate analysis showed that AI‐based models achieved a pooled sensitivity of approximately 0.78 and a specificity of approximately 0.70. In comparison, human‐based approaches demonstrated slightly lower sensitivity (≈0.74) and notably lower specificity (≈0.56). The SROC curves revealed a more compact clustering of AI studies, indicating a more consistent diagnostic performance, whereas human studies were more widely dispersed, reflecting greater variability.

## 4 Discussion

This systematic review and meta‐analysis is the first to comprehensively and rigorously assess the role of AI‐based models in predicting MT in OED, in comparison with human‐based evaluation. In line with previous reports [58], our findings highlight the considerable complexity of oral cancer prediction, reflecting the diversity of OPMDs, heterogeneity in classification systems, and variability in analytical approaches. Within this context, AI‐based models demonstrated not only strong discriminative performance but also greater consistency across studies, suggesting a potential role in stabilizing diagnostic assessment rather than simply increasing detection rates.

This review also underscores the expanding body of research aimed at developing robust RPMs for OED, with increasing incorporation of AI‐based methods. Moreover, identifying overarching trends and synthesizing the best‐performing approaches may help guide future research in this field. From 2020 onwards, the emergence of AI‐based models has signaled a progressive shift toward computational pathology. However, the current evidence base remains geographically concentrated in Europe and may not fully reflect the global landscape, particularly given that MT rates vary significantly across different continents [59]. Encouragingly, multicentric and intercontinental collaborations have become more frequent in recent studies [13, 50, 56], which may enhance the generalizability and external validity of future models.

All included studies used morphological analysis of H&E‐stained histopathological images of OED as the primary data source. In human‐based studies, 58.6% of OED cases were associated with OL, which may present as homogeneous or nonhomogeneous lesions. OED cases associated with erythroleukoplakia and erythroplakia were included in this review due to their prevalence and overlapping clinical presentations, encompassing white, speckled, and red components [60]. However, future studies should more consistently report the clinical origin of OED, as different OPMDs may be associated with distinct biological behavior and risk of MT [61]. This variability in lesion type may also contribute to between‐study heterogeneity and complicate direct comparisons across studies. Improved standardization in reporting clinical context, alongside histopathological findings, may enhance the interpretability and generalizability of both human‐ and AI‐based models.

Four different OED classification systems were identified across human‐based studies, reflecting substantial variability in histopathological assessment. Despite this heterogeneity, the meta‐analysis demonstrated a consistent and significant association between OED and MT across classification approaches, with no significant differences between subgroups (*p* = 0.183). These findings suggest that two‐tier and three‐tier systems provide broadly comparable discriminatory performance for MT risk stratification.

However, the substantial heterogeneity observed (*I* ^2^ = 85%) likely reflects not only differences in study design, diagnostic criteria, and minimum follow‐up but also inherent limitations of histopathological grading, including interobserver variability and subjective interpretation, suggesting that variability is intrinsic to human grading rather than study‐specific. Importantly, the direction of effect remained consistent across studies, with higher‐grade dysplasia consistently associated with an increased risk of MT, supporting the robustness of histopathological grading despite methodological variability. At the same time, this variability highlights a key limitation of conventional assessment, reinforcing the need for more standardized and reproducible approaches.

AI‐based studies employed diverse RPMs, including ML, DL, and hybrid approaches, generally demonstrating strong discriminative performance (pooled odds ratio [OR] = 12.64; 95% CI: 5.65–28.26; *I* ^2^ = 57%). However, methodological heterogeneity across studies limits direct comparability and raises concerns regarding the robustness and reproducibility of these findings. A notable trend is the integration of multimodal data into AI models for OED, incorporating clinical variables [54, 55, 56, 57], cytological and architectural features [54], lymphocyte scores [51, 53, 55], and DNA content [56]. While this approach may enhance predictive performance, it also introduces additional complexity and increases the risk of overfitting, particularly in studies with limited sample sizes and a lack of external validation.

Furthermore, concerns regarding transparency and interpretability remain a significant barrier to clinical adoption. As highlighted by Alajaji et al. [27], the “black box” nature of many DL models may limit trust and acceptance among clinicians. In addition, adherence to standardized reporting frameworks for AI‐based research (e.g., TRIPOD‐AI, CONSORT‐AI, and SPIRIT‐AI) was not systematically assessed in this review and was reported in only one study [56], representing an additional limitation. Future studies should follow established reporting guidelines to improve transparency, reproducibility, and clinical applicability. Despite these challenges, AI‐based models demonstrated more consistent performance across studies, with lower heterogeneity compared to human‐based approaches, suggesting a potential role in reducing variability in histopathological assessment. As these models become more refined and standardized, their clinical applicability is likely to expand, supported by their potential for broader accessibility and cost‐effectiveness.

Across analyses, AI‐based systems demonstrated higher specificity and more consistent overall performance, while sensitivity remained broadly comparable to human‐based classification. This pattern suggests that the primary contribution of AI lies not in increasing detection rates, but in improving decision precision by reducing false‐positive classifications. The observed heterogeneity in human studies was more pronounced and diffusely distributed, particularly for specificity, reflecting variability in diagnostic thresholds and subjective interpretation. In contrast, AI‐based studies showed more stable operating points and less dispersion, indicating more consistent and standardized decision boundaries.

Despite these findings, the overall certainty of the evidence remains limited. Most included studies were retrospective in design, with geographic concentration and substantial heterogeneity in dysplasia grading systems and outcome definitions. In addition, AI‐based studies frequently lacked external validation and were based on relatively small datasets, which may limit generalizability. Although the GRADE framework was not applied due to its limited suitability for prognostic and prediction model studies, these factors should be considered when interpreting the results and their potential for clinical translation.

Together, these findings suggest that AI contributes to reducing variability and improving the reproducibility of RPMs for MT in OED, with potential implications for more consistent clinical decision‐making and improved patient stratification.

## 5 Conclusion

This systematic review and meta‐analysis demonstrate that AI‐based models for predicting MT in OED achieve strong discriminative performance, with higher specificity and more consistent results compared with human‐based evaluation, while maintaining comparable sensitivity.

These findings suggest that AI contributes primarily to improving decision precision and reducing variability, rather than increasing detection rates. Despite current limitations, including the small number of studies and methodological heterogeneity, the integration of AI into histopathological assessment represents a promising step toward more standardized and reproducible risk prediction. Overall, AI appears to address one of the main limitations of dysplasia grading—its inherent variability—by providing more stable and reproducible decision thresholds.

## Author Contributions

**Diele Carine Barreto Arantes:** conceptualization, methodology, investigation, data curation, writing – original draft. **Marcelo Sperandio:** conceptualization, validation, investigation, data curation, writing – review and editing, supervision. **Lucas Monteiro Delgado:** methodology, software, formal analysis. **Saman Warnakulasuriya:** validation, writing – review and editing, supervision. **Marília de Marco Pinto Paiva dos Santos:** writing – review and editing.

## Funding

This work was supported by the São Paulo Research Foundation (FAPESP), Brazil (Grant No. 2025/08148‐4).

## Conflicts of Interest

The authors declare no conflicts of interest.

## Supporting information


**Table S1:** Search strategy.

**Figure S1:** Forest plots of the risk of MT by grade of OED: (A) mild (OR = 3.36; 95% CI: 1.85–6.10), (B) moderate (OR = 7.62; 95% CI: 3.91–14.85), and (C) severe (OR = 18.98; 95% CI: 10.25–35.14), all compared with no dysplasia. Random‐effects model. Squares represent individual studies and diamonds the pooled estimates.

## Data Availability

All data generated or analyzed during this study are included in this published article and its Supporting Information files.
