# Supplementary material for: Predicting Malignant Transformation in Oral Epithelial Dysplasia: A Systematic Comparison of Artificial Intelligence‐Based Risk Models and Pathologist‐Based Microscopy
Source: J Oral Pathol Med. 2026 Jul 23;55(9):1019–33. doi: 10.1111/jop.70168 (PMC13635274; doi:10.1111/jop.70168)

A

Study	Mild Dysplasia Events	Mild Dysplasia Total	No Dysplasia Events	No Dysplasia Total	Weight	Odds Ratio MH, Random, 95% CI
Bagan 2022	7	57	11	143	17.4%	1.68 [0.62, 4.58]
Chaturvedi 2020	18	42	38	422	23.3%	7.58 [3.78, 15.20]
Darling 2023	1	5	1	2	2.6%	0.25 [0.01, 8.56]
Jayasooriya 2020	1	34	0	20	3.1%	1.84 [0.07, 47.23]
Rodrigues 2025	11	70	2	84	10.4%	7.64 [1.63, 35.78]
Shearston 2019	0	30	2	152	3.4%	0.99 [0.05, 21.07]
Sperandio 2023	7	259	5	262	14.9%	1.43 [0.45, 4.56]
Tovaru 2023	4	34	2	75	8.7%	4.87 [0.85, 28.00]
Wills 2023	6	35	3	76	11.3%	5.03 [1.18, 21.49]
Zaini 2018	2	47	1	121	5.1%	5.33 [0.47, 60.27]
**Total (95% CI)**	**57**	**613**	**65**	**1357**	**100.0%**	**3.36 [1.85, 6.10]**

Heterogeneity: $Tau^2 = 0.271$; $Chi^2 = 13.49$, df = 9 (P = 0.1415); $I^2 = 33\%$
Test for overall effect: Z = 3.99 (P < 0.0001)

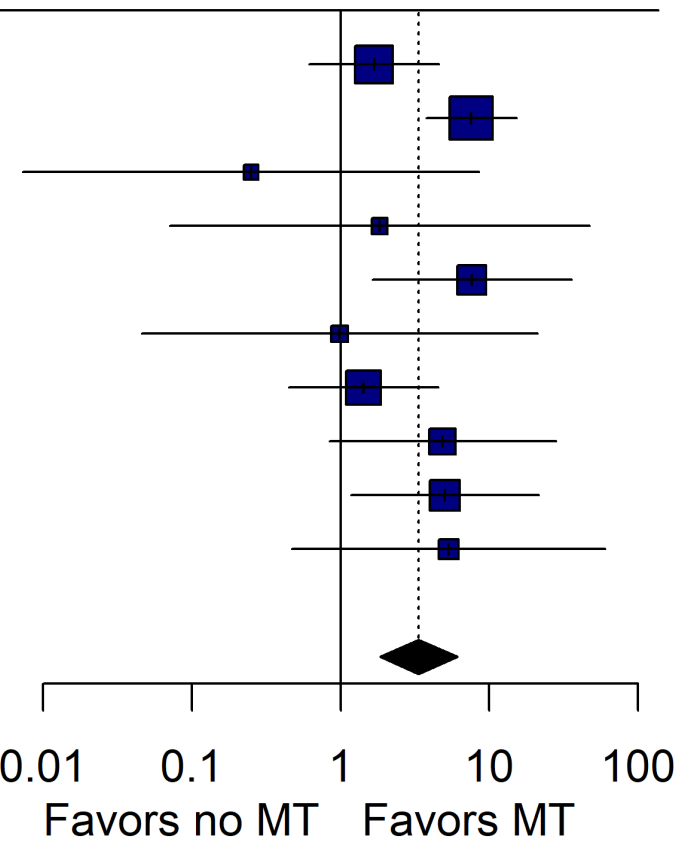

B

Study	Moderate Dysplasia Events	Moderate Dysplasia Total	No Dysplasia Events	No Dysplasia Total	Weight	Odds Ratio MH, Random, 95% CI
Bagan 2022	7	22	11	143	16.8%	5.60 [1.89, 16.62]
Chaturvedi 2020	7	16	38	422	17.5%	7.86 [2.77, 22.29]
Darling 2023	15	21	1	2	4.4%	2.50 [0.13, 46.77]
Jayasooriya 2020	1	20	0	20	3.7%	3.15 [0.12, 82.16]
Rodrigues 2025	14	29	2	84	11.2%	38.27 [7.88, 185.88]
Shearston 2019	0	8	2	152	4.0%	3.54 [0.16, 79.71]
Sperandio 2023	9	265	5	262	16.6%	1.81 [0.60, 5.47]
Tovaru 2023	3	9	2	75	8.3%	18.25 [2.54, 131.30]
Wills 2023	4	11	3	76	10.3%	13.90 [2.58, 75.05]
Zaini 2018	5	30	1	121	7.1%	24.00 [2.69, 214.41]
**Total (95% CI)**	**65**	**431**	**65**	**1357**	**100.0%**	**7.62 [3.91, 14.85]**

Heterogeneity: $Tau^2 = 0.380$; $Chi^2 = 14.06$, df = 9 (P = 0.1203); $I^2 = 36\%$
Test for overall effect: Z = 5.97 (P < 0.0001)

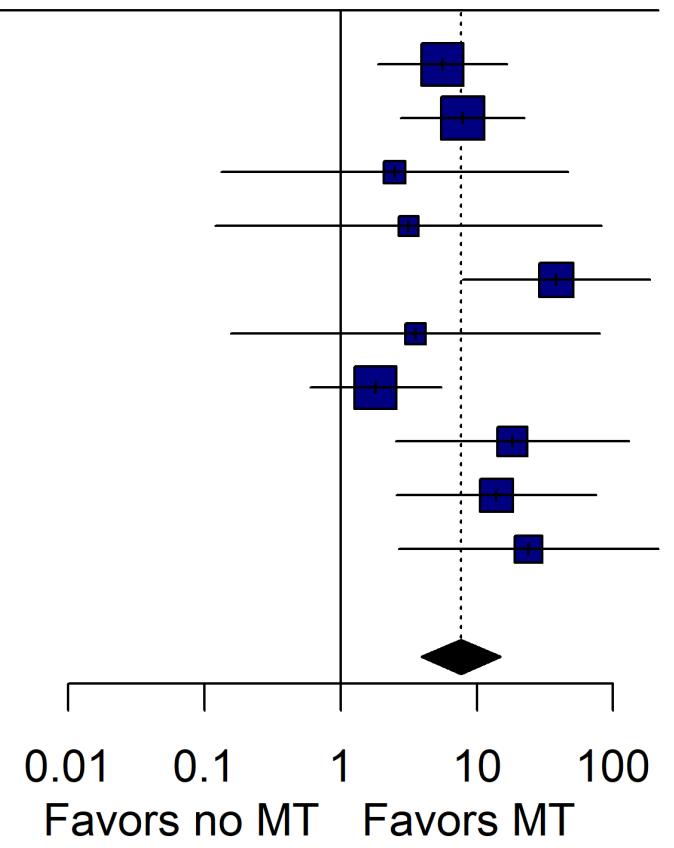

C

Study	Severe Dysplasia Events	Severe Dysplasia Total	No Dysplasia Events	No Dysplasia Total	Weight	Odds Ratio MH, Random, 95% CI
Bagan 2022	1	2	11	143	4.7%	12.00 [0.70, 205.23]
Chaturvedi 2020	8	9	38	422	8.6%	80.84 [9.85, 663.75]
Darling 2023	13	18	1	2	4.3%	2.60 [0.14, 50.05]
Jayasooriya 2020	5	19	0	20	4.3%	15.55 [0.80, 303.79]
Rodrigues 2025	7	12	2	84	11.6%	57.40 [9.37, 351.57]
Shearston 2019	1	5	2	152	5.6%	18.75 [1.40, 251.87]
Sperandio 2023	14	92	5	262	34.3%	9.23 [3.22, 26.42]
Tovaru 2023	0	2	2	75	3.5%	5.88 [0.22, 157.46]
Wills 2023	7	13	3	76	15.0%	28.39 [5.80, 138.95]
Zaini 2018	7	21	1	121	8.1%	60.00 [6.87, 524.01]
**Total (95% CI)**	**63**	**193**	**65**	**1357**	**100.0%**	**18.98 [10.25, 35.14]**

Heterogeneity: $Tau^2 = 0$; $Chi^2 = 8.75$, df = 9 (P = 0.4611); $I^2 = 0\%$
Test for overall effect: Z = 9.36 (P < 0.0001)

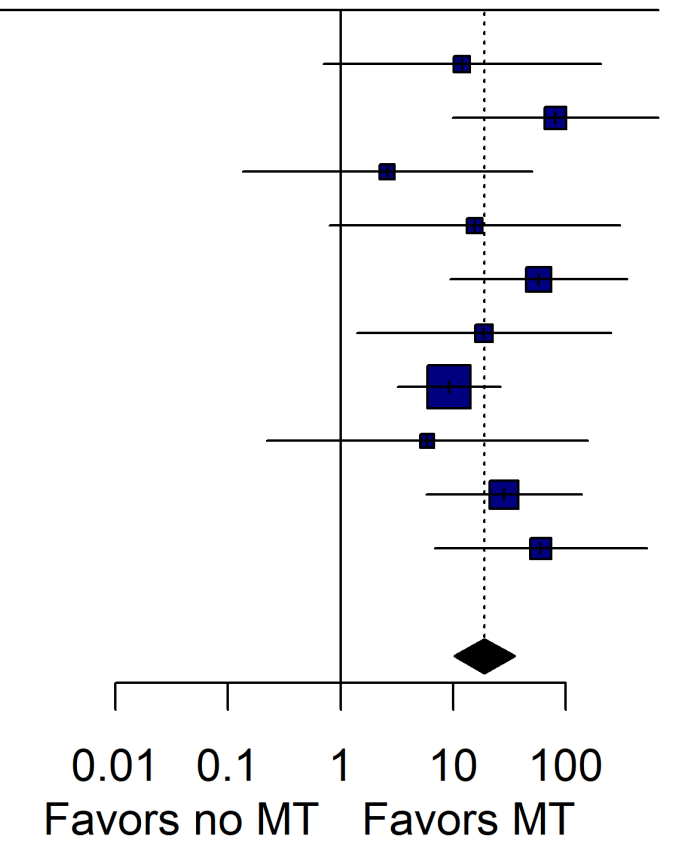

Supplement: Supplementary file 1 [file JOP-55-1019-s001.pdf]
