# Supplementary material for: Predicting Malignant Transformation in Oral Epithelial Dysplasia: A Systematic Comparison of Artificial Intelligence‐Based Risk Models and Pathologist‐Based Microscopy
Source: J Oral Pathol Med. 2026 Jul 23;55(9):1019–33. doi: 10.1111/jop.70168 (PMC13635274; doi:10.1111/jop.70168)
Supplement: Supplementary file 2 [file JOP-55-1019-s002.docx]

**Supplementary Table S1**. Search strategy

| **PubMed**  **Embase**  **Cochrane**  **ACM Digital Library** | (“Oral” OR "Mouth" OR "Oral Mucosa*” OR "Mouth Mucosa*” OR "Oral Epitheli*” OR "Oral Mucosa Epitheli*” OR “Head and Neck") AND ("Dysplasia" OR “Oral Dysplasia” OR “Epithelial Dysplasia” OR "Oral Epithelial Dysplasia” OR "Oral Epithelial Lesion*” OR “Oral Mucosal Lesion*” OR "Oral Dysplastic Lesion*” OR “Leukoplakia" OR "Proliferative Verrucous Leukoplakia” OR "Erytroplakia" OR "Erytroleukoplakia" OR "Actinic Cheilitis" OR "Oral Submucous Fibrosis" OR “Precancer*” OR “Precancer* Lesion*” OR “Pre-cancer*” OR “Pre-cancer* Lesion*” OR “Oral Dysplastic Precancerous Lesion*” OR "Premalignant" OR “Premalignant lesion*” OR “Mucosal Malignant" OR "Malignant Transformation" OR "Potentially Malignant" OR “Pre-malignant" OR “Pre-malignant lesion*” OR “Epithelial Premalignant Lesion*” OR "Potentially Malignant Lesion*” OR "Potentially Malignant Disorder*” OR “Carcinogenesis") AND ("Deep Learning" OR "Neural Network*” OR "Artificial Neural Network*” OR "Machine Learning" OR "Artificial Intelligence" OR “Automat*" OR “Automat* Method" OR “Automat* Technique" OR "Algorithm" OR “Predict*” OR “Predict* Model" OR "Risk Predict*” OR “Risk of Malignan*” OR "Cancer Predict*” OR "Oral Cancer Risk" OR "Inter And Intra-Observer" OR "Inter-Observer" OR "Intra-Observer") AND (“Detect*" OR “Detect* Technique" OR “Cancer Early Detect*” OR "Early Detect*” OR "Diagnosis" OR "Early Diagnosis" OR “Cancer Early Diagnosis" OR “Classificat*" OR “Grad*" OR “Imag*" OR “Segmentat*” OR "Whole Slide Image" OR “WSI" OR “WSI analysis” OR “Histopatholog*" OR “Histolog*" OR “Patholog*" OR "Hematoxylin And Eosin" OR “H&E”) NOT ("systematic review” OR “review*” OR “meta-analysis” OR “animal*”) |
| --- | --- |
| **IEEE Xplore** | “Oral Dysplasia” AND (“Oral Epitheli*” OR “Head and Neck”) AND (“Deep Learning” OR "Machine Learning” OR "Artificial Intelligence”) AND “Histol* Imag*” |
